# Living With and Beyond Cancer With Comorbid Conditions: Qualitative Insights to Understand Psychosocial Support Needs

**DOI:** 10.1111/hex.70039

**Published:** 2024-10-06

**Authors:** Debbie Cavers, Sarah Cunningham‐Burley, Eila Watson, Elspeth Banks, Christine Campbell

**Affiliations:** ^1^ Usher Institute University of Edinburgh Edinburgh UK; ^2^ Faculty of Health and Life Sciences Oxford Brookes University Oxford UK

**Keywords:** cancer multimorbidity, cancer survivor, comorbidity, integrated care, qualitative, survivorship care

## Abstract

**Introduction:**

There is a pressing need to understand and explore the complex experiences and psychosocial support needs of people LWBC‐CM and their informal caregivers, to inform survivorship and supportive care interventions.

**Methods:**

In‐depth qualitative interviews were conducted with people LWBC‐CM and their informal caregivers in Scotland, invited via primary care. One‐to‐one, face‐to‐face interviews were conducted with informed consent exploring experiences of symptoms, psychosocial support needs and interactions with health services. Interviews were transcribed and analysed using a thematic approach.

**Results:**

Forty‐one people LWBC‐CM and twenty‐three informal caregivers were interviewed. Four themes were identified: the Physical and Psychological Impact of Cancer and Comorbidity, Dominant Storie—Prioritising Conditions and Making Sense of Illness, Navigating Health Services and Treatments and Caring for People with Complex Health Conditions. Type and severity of conditions mediated people's experiences and daily living. Complex fatigue—fatigue arising from a number of health conditions—dominated symptomology. Participants navigated multiple appointments and complex medication regimes. Patients identified the need for acknowledgement of other chronic conditions and for streamlined care provision. Mutual caring and social isolation were also identified as part of the caring relationship.

**Conclusions:**

There is a mandate to address the psychosocial support needs of people LWBC‐CM, and their informal carers, given the burden of treatment for cancer survivors with moderate to severe complex conditions as they navigate health services.

**Patient or Public Contribution:**

A patient representative has been involved in all stages of the study from development of the application through study design, commenting on documentation, analysis of transcripts and writing the manuscript. They are included as an author on the manuscript.

## Practitioner Point

- Existing work to expand cancer survivorship models of care to incorporate other chronic conditions, and integrated models of care for multimorbidity, can work together to create coherent, patient‐centred quality provision of care for cancer survivors.

## 1 Introduction

People living and with and beyond cancer (LWBC) are now more likely than not to be dealing with other long‐term health conditions [1, 2, 3, 4]. An ageing population combined with early detection and treatment advances have contributed to the increasing prevalence of people LWBC, estimated to reach four million in the UK by 2030 [5]. Cancer survivors report poorer physical and mental health outcomes than those without cancer, with the poorest negative outcomes associated with cancer multimorbidity (two or more conditions) and being within 1 year of their cancer diagnosis [3, 6]. There is also a social gradient with multimorbidity onset (including cancer) 10–15 years earlier in people living in more deprived areas [2, 7].

The majority of follow‐up in survivorship care is currently led by secondary care [7]. Historically, there was a single disease‐focused model of care centred on the concept of cancer surveillance across the continuum from prevention to palliative care. More recently, there has been a consistent evidence‐ and theory‐driven call for a more holistic approach to survivorship care with primary care at the helm [7, 8, 9, 10, 11, 12].

In the UK, policy and initiatives such as the National Cancer Survivorship Initiative and Scotland's *Beating Cancer: Ambition and Action* and the subsequent *Transforming Care After Treatment* have identified key areas of importance in supporting people LWBC, which include a focus on health and well‐being after treatment; personalised care planning and tailored support; supporting self‐management; and recording and responding to Patient Reported Outcomes Measures (UKNCSI; Scottish Government 2016). Tailoring care to individual cancer survivors, as part of the NHS vision for personalised care, should therefore take into consideration existing and developing chronic health conditions. For the purposes of this research, cancer is regarded as the index condition and comorbidity describes any additional chronic condition reported by participants; this is also described as cancer multimorbidity in the literature [7]. There is a lack of consensus definition surrounding multimorbidity (with global variations and differences in the types of chronic conditions included), making it difficult to measure the burden and thus plan context‐specific interventions accordingly [13]. Certain conditions such as hypertension, coronary artery disease and diabetes mellitus are more commonly comorbid with cancer, and are more common in cancer survivors than in those without cancer [14]. This paper focuses on self‐reported physical and mental health chronic conditions, where chronic conditions loosely refer to the Center for Disease Control and Prevention definition of conditions that last for 1 year or more and require ongoing medical attention or limit daily activities, or both [15].

At present, not enough is known about LWBC with additional chronic health conditions (LWBC‐CM). Recent research, including a systematic review conducted by our research team, has identified a gap in the evidence base focusing on lived experience of cancer multimorbidity and highlighted the need for further qualitative work [16, 17]. The National Cancer Research Institute (NCRI), in partnership with the James Lind Alliance, have highlighted research priorities for people LWBC and researching complex health needs, to include LWBC‐CM, has also been identified as a priority [18]. In‐depth insights into the patient narrative from qualitative interviews with patients and their informal carers can provide valuable evidence to identify unmet psychosocial needs and inform provision or expansion of current holistic supportive care interventions. As such, we aimed to explore patients' experiences of LWBC‐CM and their supportive care needs.

## 2 Materials and Methods

This paper reports on qualitative interviews with patients LWBC‐CM, and their informal carers, building on prior research prioritisation work and a qualitative systematic review [16, 19]. Carer perspectives were sought to provide valuable insight into the issues facing patients, as well as those facing the carers themselves. Consolidated Criteria for Reporting Qualitative Research (COREQ) guidance was consulted to ensure rigour in our approach.

### 2.1 Identifying, Sampling and Recruiting Participants

Participants were men and women over 18 LWBC‐CM from four Scottish health board regions, and their informal carers, recruited via their general practice with assistance from the National Research Scotland Primary Care Network (NRS PCN). Participants were purposively and iteratively sampled to explore diverse experiences according to age, gender, geographical area (urban, rural and deprived locations), variation in cancer and chronic illness types and time since diagnosis of cancer. Regional coordinators from the NRS PCN reviewed patient lists and identified patients LWBC‐CM who met sampling criteria—a list of eligible comorbidities drawn from Barnett et al. [2]. A batch of recruitment packs was sent out to eligible patients by the NRS PCN coordinator, on behalf of their GP, including a cover letter, a reply card and a summary information sheet. Interested people were invited to return the reply card to the researcher, who would follow this up with a telephone call to discuss the study and establish rapport. A full information sheet (developed in partnership with patient representatives) was posted out to those who agreed to take part before interviews were arranged. Permission was asked to speak with their main informal carer (referred to as ‘the main person supporting you at home, if relevant’), information sheets were provided and interviews were arranged to coincide with patient interviews where possible. Separate interviews were conducted unless otherwise requested by participants.

### 2.2 Interviews and Consent

In‐depth, patient‐led interviews were conducted in patients' and carers' own homes or a private meeting room at the university, if preferred (the research was carried out before the covid‐19 pandemic). Telephone interviews were also conducted where preferable. All interviews were recorded with an encrypted digital recorder. Interviews were carried out by DC (PhD), an experienced qualitative health services researcher with a background in Health Psychology. Interviews lasted approximately 1 h and explored patients' experiences of illness and health care, the impact of their conditions on their daily lives, whether the timing of their diagnosis influenced their experience and how they felt about seeing different professionals for different conditions. Carer interviews covered these topics from their perspective as well as exploring carers' own needs, identified as a key priority during the research prioritisation component [19]. Written consent was obtained before interviews took place; consent forms were posted with a pre‐paid reply envelope to those taking part in telephone interviews.

### 2.3 Analysis

Interviews were transcribed and uploaded to QSR NVivo version 11 for thematic analysis, using Braun and Clarke's prescribed method, chosen to align with the exploratory nature of the study and interpretivist framework [20]. Interviews were transcribed by a third‐party contractor using a secure portal in ‘intelligent verbatim’ format. Transcripts were read repeatedly by DC to aid familiarisation, and a selection of transcripts was read by CC and by EB, who provided a patient perspective on the evolving analysis. A subset of transcripts was subjected to open coding to establish key aspects of each interview, making full use of the data set and discussed with all members of the research team, to ensure a comprehensive and rigorous analysis. These were drawn together across transcripts to form common codes or themes, to inform the development of a thematic framework. Using QSR NVivo version 12 (www.qsrinternational.com), this was applied back across the whole data set and new codes were iteratively formed as further common themes became apparent. Codes were further refined to generate overarching themes that interpreted and explained the data, reported below. Reflexive practice was ensured by memo writing to inform analysis and reflect on the role of the researcher to surface assumptions that could shape the findings.

## 3 Results

Forty‐one patient (20 females and 21 males; aged 31–82 years, mean age 65 years) interviews were carried out, and 23 interviews with informal carers were carried out in four Scottish health board regions. Fifteen LWBC interviewees lived alone and the remainder lived with a partner and/or children. Five informal carer interviews were held jointly with the patient and the remainder were carried out separately. Full details of patient characteristics are shown in Table 1.

**Table 1:** Patient characteristics.

Study ID^a^	Age/gender	Region	Cancer type	Self‐report comorbid conditions	Carer interview/interview details/living status
1	72/F	Lothian	Bowel	Hearing loss, arthritis, hypertension	Yes, separate, husband
2	69/F	Lothian	Lung, kidney	Osteoarthritis, hypertension, chronic kidney disease, benign brain tumour, skin lesions	Yes, separate, husband
3	69/M	Lothian	Chronic lymphocytic leukaemia	Severe asthma/COPD, nasal polyps, depression, digestive problems	Yes, joint, sister, lives alone.
4	82/F	Lothian	Lung	Peripheral vascular disease, COPD, angina, hypertension, dyspepsia	No, husband not available
5	76/M	Tayside	Kidney	Type 2 Diabetes, hypertension, high cholesterol, arthritis	Yes, joint with partner, lives alone.
6	71/M	Tayside	Kidney	COPD, liver disease	Yes, joint, wife
7	53/M	Lothian	Non‐hodgkin's lymphoma	Asthma, type 1 diabetes	No, lives alone, single
8	75/M	Lothian	Prostate, Acute Myeloid Leukaemia	Hypertension	No, lives alone, separated.
9	67/M	Tayside	Non‐Hodgkin's Lymphoma	Arthritis, hypertension, dyspepsia, memory problems	No, lives alone.
10	74/M	Tayside	Kidney	Hypertension, high cholesterol	No, wife not available.
11	82/F	Lothian	Kidney	Migraine, HIV, COPD, irritable bowel and bladder	No, lives alone, divorced.
12	38/F	Lothian	Breast	Anxiety, depression, chronic pain, PCOS	Yes, separate, lives with husband.
13	73/F	Lothian	Endometrial	Gout, hypertension, chronic back pain	Yes, separate, lives with husband.
14	70/M	Lothian	Prostate	Hypertension	No, divorced, lives with adult son.
15	74/F	Lothian	Hodgkin's Lymphoma	Ulcerative colitis, ‘weak chest’ (?COPD)	Yes, separate interview with daughter, widowed; lives alone.
16	61/F	Lothian	Breast	Crohn's disease, rheumatoid arthritis	No, lives alone; partner not available.
17	70/M	Lothian	Leukaemia	Hepatitis B, mild memory and language processing difficulties (associated with an accident)	Yes, separate, lives with wife.
18	73/M	Lothian	Bowel	Gout, Type 2 diabetes, arthritis	Yes, joint with friend, widowed, lives alone.
19	77/M	Lothian	Non‐Hodgkin's Lymphoma	Heart disease, high cholesterol	No, partner not available, lives alone.
20	79/M	Lothian	Bladder, bowel	Heart disease, Type 2 diabetes, hypertension, memory problems	Yes, joint, lives with wife.
21	60/F	Lothian	Breast	Asthma, endometriosis, anxiety, depression	Yes, separate, lives with partner.
22	55/F	Lothian	Breast	Type 1 diabetes, chronic back pain, overactive thyroid, trigger finger, aortic stenosis.	Yes, separate, lives with husband
23	77/F	Lothian	Breast	Ulcerative colitis, hypertension, underactive thyroid, rheumatoid arthritis	Yes, separate, lives with husband.
24	57/F	Lothian	Breast	Hypertension, endometriosis	Yes, separate, lives with husband.
25	71/M	Lothian	Throat	Angina, high cholesterol, asbestosis	Yes, separate, lives with wife.
26	Late 50s/F	Lothian	Breast	Hypertension	No, husband not available, lives with husband.
27	66/F	Lanarkshire	Oral	Depression, hypertension	Yes, separate, lives with husband.
28	68/M	Lanarkshire	Prostate	Rheumatoid arthritis	Yes, separate, lives with wife.
29	68/F	Lanarkshire	Breast	Asthma, anxiety, depression, haemochromatosis, diverticulitis, type 2 diabetes, underactive thyroid.	Yes, separate interview with sister, lives alone.
30	67/M	Lanarkshire	Myeloma	Type 1 Diabetes, Crohn's disease	Yes, separate, lives with wife.
31	62/M	Lanarkshire	Prostate	Anxiety, depression	No, single, lives alone.
32	73/M	Lanarkshire	Leukaemia	Heart disease, hypertension, underactive thyroid	No, partner not available, lives alone. Telephone interview.
33	Mid‐late 60s/M	Tayside	Tonsil	Type 2 diabetes	Yes, separate, lives with wife. Telephone interview.
34	79/M	Tayside	Prostate	Spinal stenosis, chronic pain, glaucoma, arthritis, scoliosis, diverticulitis, stomach problems.	Yes, separate, lives with wife. Telephone interview.
35	72/F	Tayside	Cervical, breast	Arthritis, hypertension, underactive thyroid	No, lives alone, divorced. Telephone interview
36	77/F	Tayside	Oesophageal	Arthritis, hypertension, bowel disease, dyspepsia	Yes, separate, lives with husband. Telephone interview.
37	64/M	Tayside	Bladder	COPD	No, partner not available, lives with partner. Telephone interview.
38	31/F	Tayside	Breast	Anxiety, depression	No, partner not available, lives with partner and young daughter. Telephone interview.
39	60s/F	Dumfries and Galloway	Breast	Depression	No, lives alone. Telephone interview.
40	73/F	Dumfries and Galloway	Breast	Hypertension	No, husband not available, lives with husband. Telephone interview.
41	70s/M	Dumfries and Galloway	Prostate	ME	Yes, joint, lives with wife. Telephone interview.

a Study ID does not necessarily correspond to interview number, as some interviews were conducted separately with carers.

The most common types of cancers experienced by participants were breast, blood cancers, prostate, kidney, bowel and lung. The most common chronic conditions were high blood pressure, arthritis, diabetes, anxiety and depression, bowel disease, heart disease, COPD, asthma and chronic pain. Most of these conditions were diagnosed before the cancer. Nine people had cancer and one other condition; the remaining 32 people had two or more additional self‐reported conditions.

Four key themes were identified to capture people's experiences of LWBC‐CM: *the Physical and Psychological Impact of Cancer and Comorbidity, Dominant Stories—Prioritising Conditions and Making Sense of Illness, Navigating Health Services and Treatments and Caring for People with Complex Health Conditions*.

### 3.1 The Physical and Psychological Impact of Cancer and Comorbidity

The cumulative burden of conditions and symptoms had a corresponding growing effect on people's ability to deal with and manage their health.

Oh God, what's this now, another thing to cope with, another thing to manage.

*(Int 20, 70yo M, leukaemia)*

The tally of conditions themselves was not the issue; rather, participants reported that it was more the burden of symptoms. This could mean that one person who had experienced an early‐stage cancer that has been treated successfully with minimal side effects and have medically managed high blood pressure may feel little impact of their conditions or not even consider themselves as ill. On the other hand, a person with ongoing side effects of cancer treatment as well as not well‐controlled diabetes or COPD and a mental health condition such as depression experienced a daily burden of symptoms that dominated day‐to‐day living and overshadowed their personal identity. Illness came to be expected among the older interviewees—‘add it to the list’ as one participant commented. For those with a heavy burden of symptoms, their lives were impacted considerably and this led to significant adaptations to daily living and, for some, increased social withdrawal and isolation:

I used to be like a ball of fire, you know. You wouldn't believe what I used to do in 1 day. Because I was into everything. But, unfortunately, them days have gone now.[…] I'm just up here wasting away, you know.

*(Int 13, 67 yo M, non‐Hodgkin's lymphoma)*

Fatigue—an extreme lack of energy and motivation (attributed to multiple conditions)—was a common symptom, along with brain fog and chronic pain. This can be understood as ‘complex fatigue’, as it is intertwined with other symptoms and results from a cumulative burden of health conditions impacting on people's lives, as shown in the examples below:

It just makes you really, really tired, and my joints have been sore, really sore. So I'm now seeing another consultant, because they think I maybe have rheumatoid arthritis, but that can come from your Crohn's as well, seemingly.

*(Int 15, 61yo F, breast cancer)*

Some days I just feel so tired. And I still have to go and lie down, you know, I just…Washed out, yeah. And I used to do at least three to five miles every day, and lately, I just can't do it, you know.

*(Int 18, 76 yo M, kidney cancer)*

There was also an interplay between conditions that made it difficult to attribute symptoms to any one condition and also impacting on recovery following cancer treatment, as was the case for this participant with kidney cancer and COPD:

COPD …just gets worse if you're inactive. And that was the big effect of the [cancer] operation, it was nothing to do with cancer, or the aftermath of the operation. It was just, the COPD got worse, and it's irreversible, so.

*(Int 6, 71 yo M, kidney cancer)*

### 3.2 Dominant Stories—Prioritising Conditions and Making Sense of Illness

There was a great deal of variation in people's narratives in terms of what dominated their accounts. The story most commonly told was of the condition that participants perceived had the biggest functional impact on their everyday lives:

I find the shoulder and back pain define who I am more than the other conditions because […] the pain maybe does…I still probably do as much as I did but rather I feel it defines me.[…] It is exhausting.

*(Int 26, 55 yo F, breast cancer)*

Cancer left a lasting legacy on people's lives and narratives around cancer continued to dominate regardless of the severity of the disease and its physical impact. In certain cases, the physical impact of cancer was often encapsulated in time for people surrounding treatment and recovery. Here, cancer became less dominant over time, particularly when eclipsed by the limitations of other, more chronic health conditions with debilitating symptoms such as COPD, heart disease, diabetes and mental ill health. In this sense, cancer was not always the priority:

The diabetes could kill me on a daily basis, I get up to have my injection, if I forget to eat or if I fall asleep, I'm dead basically.

*(Int 10, 53yo M, Hodgkin's lymphoma)*

However, cancer appeared to have a distinct psychological and shared social meaning. The diagnosis of cancer was a pivotal moment with a devastating emotional impact on many patients and carers interviewed but not necessarily the biggest disruption or change to physical functioning on a longer term basis, as described by this participant and his wife:

Well the biggest shock was the kidney removal [cancer]. But that hasn't had a big impact.

*(Int 11, 74yo M, kidney cancer)*

R2:If somebody said that word [cancer] to me, honest to god, it would be like a death sentence.

*(Int 6, carer)*

This highlights a distinction between psychological impact and physical impact of illness and the complex interplay between them. Moreover, the emphasis is on the disruption caused by the burden of symptoms rather than a specific condition. This complexity of experience is further influenced by the severity and trajectory of a particular condition over time: one of gradual decline (such as COPD) versus static and well‐controlled (e.g., hypertension).

A cancer diagnosis was also distinct in the sense that it brought a lasting fear of recurrence when compared to other, more insidious conditions.

The cancer was a shock and that really kind of rocked our boats and made you very aware of your own mortality—so that's a life‐changer and I don't think that anxiety ever goes away now.

*(Int 25, carer)*

### 3.3 Navigating Health Services and Treatments

For those with multiple symptomatic conditions, there is heavy and repeated contact with services. Participants described experiences of multiple appointments, often in different locations and with little apparent coordination between them. Certain conditions, such as arthritis, thyroid or hypertension, were managed by GPs while others, like heart and lung disease, required specialist input and regular visits to hospital clinics. For many, this was perceived as manageable, and cognitively evaluated as being reasonable and expected in the context of multimorbidity:

I mean it's not like every second week I'm going to a clinic; there are some times you do get one or two come at the same time, but that's nothing really.

*(Int 24, 79 yo M, bladder and bowel cancer)*

Participants like this one presented themselves as very tolerant of busy schedules and medication regimes, accepting this as part of their ‘health work’.

Between my diabetes and my cancer, I've been going several times a year but you just have to live with it.

*(Int 10, 53 yo M, non‐Hodgkin's lymphoma)*

A number of participants reported that follow‐up was focused on only the condition at hand, with the exception of considering the impact of other conditions on surgery. Participants felt that specialists did not take account of their other conditions or discuss them routinely. The onus was on participants to coordinate their multiple conditions and related medications—polypharmacy was common. This participant suggested having one professional to oversee care holistically:

[It] is understandable in a way in terms of the way the NHS is structured and funding and everything but it would be nice to have one person who looked at everything. […] Because simple things like the last one I had to tell the doctor that I was Type 1 before he did something and, yeah, I wasn't sure that my notes had even been looked at.

*(Int 26, 55 yo F, breast cancer)*

Overall, participants reported that they valued seeing a specialist who they perceived as understanding a specific condition best, even if this meant multiple appointments for different conditions. Although they saw the value in a ‘one‐stop shop’ generalist clinic, many participants did not think that this was a possible option to meet their holistic needs and viewed it as difficult to coordinate in times of busy, overstretched health services:

I'd rather see the ones that know the nitty‐gritty of that particular [condition].

*(Int 31, 73yo F, endometrial)*

That would be a dream come true if we could do it very efficiently. It's understandable it's not… I think I prefer her going to experts in each of the individual fields rather that it being a sort of one‐stop shop.

*(Int 25, carer)*

Probably they can't do it, they're too busy. It must've been easier years ago, for the same consultant to be there all the time. But they've got that many things to do nowadays, and they're in demand, you know, so whether it's possible.

*(Int 1, 72yo F, bowel cancer)*

Although some people reported having good relationships with their GP and regular contact, primary care was seen as a heavily strained service that lacked in continuity of care.

I: What care and support for any of your other conditions? I mean you mentioned obviously for the arthritis it hasn't really been…

P: No, there hasn't been much support for that. […] I've had investigations for the rhinitis and all that, and that's it. They just seem to…they just give you repeat prescriptions and that's it, get on with it. Maybe because they're too busy.

*(Int 1, 72 yo F, bowel cancer)*

### 3.4 Caring for People With Complex Health Conditions

Understanding the perspective of carers and their individual needs was highlighted as a priority in the related research prioritisation exercise carried out to inform the development of this interview study [19]. Carers' views and experiences have been referred to elsewhere in the findings, but there is a focus here on issues relating specifically to caring. The caring role was varied and depended on the severity and complexity of their loved ones' ill health. Dealing with the cumulative burden of conditions also took its toll on informal carers. Carers were very focused on the needs of their loved ones and minimised their own needs, which is an important finding in itself when considering support for carers. The care needs of people LWBC‐CM brought a particular set of practical and emotional challenges as people deal with the high burden of symptoms and the complexity of managing them, at times dominating carers' lives and becoming a source of distress. Most carers were spouses of a similar age and had their own health needs to manage and so, in many instances, it was a case of mutual care and support. These participants allude to the concept of mutual caring:

We pass the baton.

*(Int 26, 55yo F, breast cancer)*

It does have an impact on me, but me getting older is having an impact on him as well, so it's just, you know, you've to just work around it, and make allowances.

*(Int 6, carer of 71 yo M, kidney cancer)*

Couples became socially withdrawn together as their support needs increased:

We don't get that many visitors, we get a hell of a lot of phone calls, but we don't just get people dropping in and saying, oh, how are you feeling today?

*(Int 30, 67 yo M, myeloma)*

## 4 Discussion

### 4.1 Summary of Main Findings

Living with cancer and other chronic conditions brought an increased burden of symptoms that impacted on day‐to‐day life for a number of participants. There was a level of variability depending on the type and severity of chronic conditions. *Complex fatigue* was the most commonly reported issue, not necessarily attributable to any one health condition but heightened by the increased burden of symptoms borne from multimorbidity. Variation was also evident in terms of the dominant narrative shared by participants, dependent on which condition brought the greatest burden of symptoms and, therefore, impact on functioning. Although cancer did not always have the greatest physical impact over time, the lasting psychological impact of a cancer diagnosis was evident. LWBC‐CM brought with it increased time spent managing illness and seeing multiple specialists in different locations. Participants reported a level of acceptance of the burden of illness management. Overall, there was a preference for specialist care but improved coordination and streamlining of care would be appreciated, as would acknowledgement and discussion of other chronic conditions by health care professionals. Informal carers reported the necessity for mutual caring, as they dealt with their own health care needs. Those with complex health care needs, particularly among older participants, also reported a degree of social isolation as their health needs increased.

### 4.2 Comparison With Existing Literature and Theory

#### 4.2.1 Cancer, Comorbid Conditions and the Burden of Treatment

The increased burden of symptoms related to LWBC‐CM for patients with complex and severe conditions is evident in the broader multi‐morbidity literature [21, 22, 23]. Living with severe symptoms is more likely to impact on and disrupt people's day‐to‐day lives and their sense of self [24, 25, 26]. Along with managing multiple symptoms, complex illness also brings additional illness management responsibilities such as managing multiple appointments and medication regimes, in addition to cancer survivorship follow‐up care [27, 28]. Carl May's Burden of Treatment theory provides a model that encompasses not only the work of managing symptoms but also the resource demand of the cognitive and practical work of engaging with and navigating health services and social networks as well as the accountability of self‐management for patients and their carers [29, 30, 31]. Helping support patients with multiple conditions adds to the burden on carers also, in addition to their own health needs [23, 32]. In our study, we noted *mutual* caring and thus a shared ‘burden of treatment’. Acknowledging patient and family caring and symptom management work, capacity and context and tailoring services to support self‐management and address unmet needs (including issues such as social isolation and carers' needs [32, 33]) are therefore a necessary part of person‐centred survivorship care.

#### 4.2.2 Where Cancer and Other Conditions Feature in the Illness Story

Where cancer survivorship fits in the story of people living with multiple conditions varies depending on the stage and severity of the cancer and its treatment, as is evident elsewhere in the cancer survivorship literature [16, 17]. Debilitating symptoms, invasive treatments and long‐term and late effects have a greater impact and cancer becomes a ‘main player’ dominating people's stories. Cancer can differ from other chronic illness, such as organ failure, in terms of their trajectories, with differing illness events and peaks in symptomology and disruption to daily living and associated distress [34]. Cancer has been described as having a ‘dramatic entrance’ to one's illness narrative compared to other chronic conditions such as COPD, which can have a different meaning for patients and are considered more ‘a way of life’ [35, 36]. Charmaz talks about how people's illness experiences and meaning making are shaped by shared social constructs of illness [37]. In this way, cancer is viewed differently to other chronic illnesses, which people work to keep on the ‘margins of their lives and outside the boundaries of their self‐concepts’ (P4 [37]) for as long as possible, which aligns with the findings from our interviews. There is a temporal ‘acute’ element for many people with cancer surrounding diagnosis and treatment in a liminal phase, where life is put on hold and cancer is prioritised [17, 38]. This is in line with Parsons' conventional sick role, as a disruption to daily life is expected and accepted by those around you [39]. This phase passes as their treatment finishes and their symptoms lessen, although it is worth noting that cancer and its treatment can contribute to a deterioration in overall functional status, particularly for older adults [27, 40]. For many, this is in contrast to other chronic conditions —often requiring daily medications—that endure and continue to disrupt daily living and shape illness identity [16, 27]. However, as noted in our interviews, the cognitive and psychological impact of a cancer diagnosis and LWB cancer can remain. The meaning ascribed to being a ‘person with cancer’ and the threat to life and self appear to carry more weight than other chronic illnesses, regardless of their potential threat and impact on lives. In this way, cancer has a shared social meaning and vocabulary that continues to be woven into people's illness stories [41]. This picture is not clear cut. There is some evidence in the cancer literature exploring the long‐term impact of cancer treatment and the psychological distress associated with a cancer diagnosis, suggesting that although distress is long lasting, the biggest impact on distress and quality of life is associated with impairment or disability [6, 42]. Regardless, although cancer survivors' physical needs may diminish over time (particularly when eclipsed by the limitations of other health conditions), their need for psychosocial support to manage survivorship is likely to remain for many years, with implications for long‐term survivorship care. Also, this survivorship needs to be increasingly understood in the context of other health conditions, personal and illness identities and overlapping symptoms. Indeed, often, it can be difficult to disentangle the complex symptoms of LWBC‐CM, again placing emphasis on symptoms over disease.

#### 4.2.3 Complex Fatigue

Fatigue appeared to be a commonly threaded symptom in this study and became a debilitating condition in itself, compounded by multiple conditions contributing to the burden. Participants reported that their struggles with fatigue were often not acknowledged by health care professionals. Fatigue has been widely reported in both the cancer and multimorbidity literature, including the long‐term impact of ‘complex’ fatigue [43, 44]. The need for research into cancer‐related fatigue was highlighted as a key priority by the NCRI (https://www.ncri.org.uk/the-uk-top-10-research-priorities-for-living-with-and-beyond-cancer/) and interventions have been developed to support fatigue management such as Macmillan's RESTORE (https://macmillanrestore.org.uk/home). The benefits of physical activity in mediating long‐term fatigue have been noted in recent years, which could have utility in managing complex fatigue in patients LWBC‐CM [45, 46, 47].

### 4.3 Strengths and Limitations

Our study has provided rich, in‐depth insight into LWBC‐CM and increased our understanding of the variability and individuality of complex conditions, with implications for person‐centred illness management. This study was conducted with NHS patients living in Scotland, UK, and can contribute to the growing evidence base on this topic.

There is reason to interpret some of the findings from our study with caution and in the context in which the data were generated. For example, our study population was in relatively good health at the time of the interview—a number of participants were living with well‐managed hypertension or with conditions where pain was well controlled as well as their cancer—and their views may differ from those with recurrent or metastatic cancer or advanced chronic conditions. People with more profound long‐term or late effects or higher level of disability or impairment as a result of their illnesses may also have different experiences. We took steps to recruit a diversity of people and included practices in urban areas of deprivation across four health boards. However, those with a higher burden of illness may not have decided to take part and, as such, their views may be missing. Research focused on LWBC‐CM with advanced diseases is warranted. We were also unable to explore issues related to ethnicity due to the lack of ethnic diversity among respondents.

We undertook to speak to informal carers about their experiences and needs. However, carers were reluctant to discuss their own needs, perhaps related to the all‐encompassing nature of caring or a perception that ‘the patient should come first’, and thus our data set is limited in findings on carer perspectives. Carer needs in caring for people LWBC‐CM are also a research priority going forward.

### 4.4 Implications of Findings and Future Research

Models of holistic and integrated care are discussed below and key points and recommendations are summarised in box 1.

Box 1: **Key Points and Recommendations**

- Shift to a symptoms focus of patients' experience of LWBC‐CM over a disease focus, with a holistic approach targeting symptom management.
- Acknowledgement of mutual caring and a holistic approach to supporting patient and carer in each role as a dyad.
- Focus on coordinating specialisms to provide shared care to patients with multimorbidity.
- Further evaluation of a navigator role to support patients in self‐management and engaging with multiple services.

#### 4.4.1 Models of Holistic Care

Primary care is often at the centre of discussions around management for both cancer survivorship and multimorbidity, and there is evidence for higher consultation rates in these groups compared to controls [7, 48]. Consideration should be given to a patient‐centred approach to holistic primary care as LWBC‐CM becomes the norm for general practitioners caring for those LWBC [49, 50]. In addition to managing symptoms, there is scope for managing the psychosocial aspects of LWBC‐CM in primary care as part of a holistic model of care [7]. Adam and Watson highlight four dimensions to support people LWBC—*contact, comprehensiveness, continuity and coordination* [11]. The dimensions of comprehensiveness and coordination can meaningfully encompass comorbid conditions and the need for holistic care, not only of the patient but also of their informal carer. However, the burden on primary care is apparent as services increasingly look to them for condition management, surveillance and follow‐up [11, 51]. There is also contention reported in the literature about who is best placed to manage follow‐up, with oncologists having less confidence in a primary care approach and primary care professionals favouring a shared care or integrated approach [51, 52, 53]. There is evidence from our study that specialist knowledge is valued and well‐coordinated specialist care was preferred over a one‐stop shop with a single health care professional. Lack of communication and poorly defined roles have been highlighted as key issues preventing effective and quality care provision [51, 52]. Survivorship care plans and Health Needs Assessments have been reviewed, but at present, there is not enough evidence for their comprehensive implementation or improved patient outcomes [9, 54, 55]. Nurse‐led models of care have been investigated as a ‘point person’ to coordinate and signpost to services to meet the individual needs of cancer survivors, including managing comorbid conditions [51, 56, 57]. More recently, risk‐stratified follow‐up care has been explored in the UK to best meet the needs of cancer survivors according to the complexity of their illness, but the challenge remains of who will manage other chronic conditions under this model of care, which necessitates conversations within secondary care as well as between primary and secondary care and including patient partners [58, 59].

#### 4.4.2 Facilitating an Integrated Care Approach

Models of integrated care have been explored for a patient‐centred approach to managing people with multiple conditions that could also incorporate patients LWBC‐CM [60, 61, 62], as well as from a cancer perspective [63, 64], further suggesting a need for open communication across specialties and a shared approach. Developing a framework for quality care provision that is flexible to patients' needs is an important guiding principle, with components such as multidisciplinary working, supporting shared decision‐making, supported self‐management, training for health care providers and integrated information technology at the heart of care provision [61]. Technological advances could be adapted to monitor people with complex health conditions, such as use of remote monitoring apps [65]. However, it is essential to consider the burden placed on patients and their carers to support this such that it is tailored to individual needs. Application of models of integrated care with a nurse navigator role to oversee, coordinate care and consider the ‘total picture' requires further evaluation [66].

## 5 Conclusion

Living with multiple chronic conditions is now the norm for many people living with and beyond cancer. Understanding patients' complex experiences and support needs is essential to plan best supportive care for cancer survivors. A number of models of integrated care have been proposed from different disciplines and specialties to manage multimorbidity. Creating a unified approach that is coordinated by one ‘point person’ is essential to enable open lines of communication and optimal patient‐centred, individualised care for people as they navigate complex conditions and seek quality of living.

## Author Contributions

**Debbie Cavers:** conceptualisation, investigation, funding acquisition, writing–original draft, methodology, formal analysis, project administration, data curation, visualisation, validation, software, resources, writing–review and editing. **Sarah Cunningham‐Burley:** investigation, writing–review and editing, visualisation, mthodology, formal analysis, supervision, validation. **Eila Watson:** investigation, visualisation, writing–review and editing, formal analysis, supervision, methodology, validation. **Elspeth Banks:** investigation, writing–review and editing, formal analysis, visualisation, validation. **Christine Campbell:** investigation, writing–review and editing, methodology, validation, visualisation, formal analysis, supervision.

## Ethics Statement

Ethical approval was obtained from the NHS West of Scotland Research Ethics Committee, reference 18/WS/0112. The authors affirm that all individual participants provided written informed consent to participate in research interviews for this study and for anonymised quotes to be included in published articles and other forms of dissemination as part of the research.

## Conflicts of Interest

The authors declare no conflicts of interest.

## Data Availability

Anonymised data are available upon reasonable request to the corresponding author.
